# Potential for Raman spectroscopy to provide cancer screening using a peripheral blood sample

**DOI:** 10.1186/1758-3284-1-34

**Published:** 2009-09-17

**Authors:** Andrew T Harris, Anxhela Lungari, Christopher J Needham, Stephen L Smith, Michael A Lones, Sheila E Fisher, Xuebin B Yang, Nicola Cooper, Jennifer Kirkham, D Alastair Smith, Dominic P Martin-Hirsch, Alec S High

**Affiliations:** 1 Oral Biology, Leeds Dental Institute, University of Leeds, Leeds, UK; 2 School of Computing, University of Leeds, Leeds, UK; 3 Department of Electronics, University of York, York, UK; 4 Section of Experimental Therapeutics, Leeds Institute of Molecular Medicine, University of Leeds, Leeds, UK; 5 School of Health Studies, University of Bradford, Bradford, UK; 6 Leeds Teaching Hospitals NHS trust and University of Leeds, Leeds, UK; 7 AVACTA group plc, York Biocentre, York Science Park, York, UK; 8 Department of ENT/Head and Neck Surgery, Calderdale and Huddersfield NHS Trust, Yorkshire, UK; 9 Department of Pathology, Leeds Dental Institute, University of Leeds, Leeds, UK

## Abstract

Cancer poses a massive health burden with incidence rates expected to double globally over the next decade. In the United Kingdom screening programmes exists for cervical, breast, and colorectal cancer. The ability to screen individuals for solid malignant tumours using only a peripheral blood sample would revolutionise cancer services and permit early diagnosis and intervention. Raman spectroscopy interrogates native biochemistry through the interaction of light with matter, producing a high definition biochemical 'fingerprint' of the target material. This paper explores the possibility of using Raman spectroscopy to discriminate between cancer and non-cancer patients through a peripheral blood sample. Forty blood samples were obtained from patients with Head and Neck cancer and patients with respiratory illnesses to act as a positive control. Raman spectroscopy was carried out on all samples with the resulting spectra being used to build a classifier in order to distinguish between the cancer and respiratory patients' spectra; firstly using principal component analysis (PCA)/linear discriminant analysis (LDA), and secondly with a genetic evolutionary algorithm. The PCA/LDA classifier gave a 65% sensitivity and specificity for discrimination between the cancer and respiratory groups. A sensitivity score of 75% with a specificity of 75% was achieved with a 'trained' evolutionary algorithm. In conclusion this preliminary study has demonstrated the feasibility of using Raman spectroscopy in cancer screening and diagnostics of solid tumours through a peripheral blood sample. Further work needs to be carried out for this technique to be implemented in the clinical setting.

## Introduction

Cancer poses a massive health burden with incidence rates expected to double globally over the next decade[1]. In the United Kingdom screening programmes exist for cervical, breast and colorectal cancer with the premise that early detection affords early intervention with improved chance of cure. Screening programmes at present target a cancer within an asymptomatic sub-group of the population deemed to be at greater risk of suffering the disease. The potential to screen patients in the community through a peripheral blood sample for any solid malignant tumour would revolutionise cancer services. Patients with a positive result could then be referred for further investigation; whilst a negative result would allay anxiety and reduce the need for further expensive investigations with possible associated morbidity.

Previous work has investigated plasma and serum levels of deoxyribonucleic acid (DNA), and ribonucleic acid (RNA) in attempts to detect the presence of cancer [2-5]. It has been reported that serum and plasma samples from cancer patients generally have higher concentrations of these nucleic acids compared with control samples. The origin of the increased levels has not yet been fully elucidated but is thought to be due to a variety of different mechanisms including; apoptosis, tumour necrosis, and possibly active release. However, a large European, multi-centred, randomised controlled trial, utilising samples from patients with various types of cancer, healthy controls and patients with chronic obstructive pulmonary disease concluded plasma concentrations of DNA were neither sensitive nor specific enough for cancer diagnostics or screening purposes[6].

Fluorescence spectroscopy has also been used for potential cancer detection in blood samples [7]. These experiments suggested that differences between cancer and non-cancer cohorts may arise due to the modification of fluorophores by tumour associated metabolites, changes in protein composition, and possible changes to the environment of the fluorophores thus altering their physicochemical properties. As yet fluorescence spectroscopy has not been applied to the clinical setting in cancer detection.

This highlights the need for a sensitive biochemical analysis where changes in the biochemical composition of blood products can be detected. It may then be possible to detect the presence of cancer in an individual whether due to the presence of abnormal molecules; increased levels of naturally occurring biochemical products; or changes in native biochemistry secondary to tumour formation.

Raman spectroscopy relies on the scattering of photons when incident light interacts with the target matter. The molecular interactions cause a frequency shift that reflects the energy of particular molecular vibrations. These vibrations are molecular bond specific allowing a 'biochemical fingerprint' to be constructed of the material. Any physiological change or pathological process that results in changes to the native biochemistry would therefore lead to changes in Raman spectra. These spectra are highly detailed allowing subtle differences in biochemistry to be identified. This technique has received much attention in tissue diagnostic possibilities but there is a paucity of literature in its application to cancer diagnosis through a peripheral blood sample [8-11].

Raman spectra of biochemical compounds produce high dimensional multivariate datasets; in order to 'unlock' this data to detect subtle differences between cohorts it is paramount that the analysis is performed by equally sophisticated techniques. Multivariate statistical methods such as Principal Component Analysis (PCA) and Linear Discriminant Analysis (LDA) are generally accepted for use in the classification of Raman spectra[12,13]. PCA transforms the data into a subspace in which the major modes of variation are captured as a linear combination of the original input. LDA is a straightforward classification scheme which rather than reducing the dimensionality of the data based on how it varies, aims to efficiently select the linear combination of the input that best discriminates classification of each example to its corresponding label e.g. Cancer/Non-cancer. Along with conventional techniques a more novel approach of using genetic evolutionary programming to evolve a classifier was used in this study. This pattern recognition technique combines rules and features in order to evolve a classifier.

Whether based upon the detection of cancer products or the body's response to the disease process the advantage of cancer screening through a peripheral blood sample is clear. The aim of this preliminary study was to identify whether Raman spectroscopy coupled with sophisticated data analysis techniques could discriminate between peripheral blood samples from patients with and without cancer.

## Materials and methods

### Patient samples

All patients participating in this study did so with their full informed consent. Peripheral blood samples were obtained from 20 patients attending the Leeds Head and Neck multidisciplinary clinic. All patients were new referrals to the clinic, although this would also include patients who had previously undergone resection of cancer and had re-presented with recurrence. Patients had tumours from the head and neck; tongue, larynx, skin, and salivary glands. The tumours were of various type, grade and stage.

Control samples were obtained from patients attending the Leeds Chest Clinic. These patients suffered a range of respiratory diseases including asthma, chronic obstructive pulmonary disease (COPD), and bronchiectasis. Patients with a history of cancer or ongoing treatment for cancer were excluded from the study.

### Ethics

Ethical opinion was sought and granted from Leeds West ethical committee. Ref: 06/Q1205/226.

2 blood samples were collected from each patient in specialist blood tubes containing 3.2% sodium citrate. Samples were centrifuged immediately at 3000 rpm for approximately 10 minutes. The supernatant (plasma) was removed and split into 4 cryovials. These vials were placed in a -80°C freezer for storage until use.

### Raman spectroscopy

The Raman spectra were obtained from all samples using a Renishaw 'System 1000' Raman microscope. Excitation was provided by a Sacher Lasertechnik Littrow external cavity laser set at 783 nm. Detection of the Raman scattered light was via a Renishaw RenCam NIR enhanced thermoelectrically cooled CCD camera. The spectrometer was coupled to a Leica DMLM microscope; and the exciting light is delivered to the sample, and the scattered light collected from the sample, via a 50 times Leica microscope objective. The spectrometer used holographic notch filters to remove Rayleigh scattered light from the collected light. The Raman scattered light was then dispersed across the CCD array detector by a single stage, 250 mm focal length grating spectrometer. The microscope was equipped with a motorised XYZ positioning stage (Prior) with integrated position sensors on the X and Y axes (Renishaw). Instrument control and data collection were performed with Renishaw WiRE software which operates within Galactic GRAMS software.

### Raman measurements

Individual samples were each thawed and pipetted onto a quartz microscope slide. This was allowed to air dry before Raman spectroscopy measurements were taken. An extended spectrum reading from 600 - 1800 nm was recorded. The time to acquire the spectral reading was 20 seconds for all samples. The microscope lens was a 50 times air objective with a 0.75 numerical aperture. Ten spectra were obtained from each plasma sample. The 10 spectra could then be converted to a mean spectrum for each sample. The plasma samples for the cancer and non-cancer patients were run alternate so as to rule out any possible influence of time of day and machine variability.

### Data normalisation

The intensity of each Raman spectrum not only depends on sample characteristics but also on operating equipment. The equipment currently used was un-calibrated as an experimental calibration protocol has yet to be devised. The raw data was normalised in order to compare data across samples as follows;

• The data was re-sampled so that the measurements all correspond to the same Raman shift points (601, 602,,1800) by interpolation between the closest points.

• Each spectra is normalised so that the area under the curve between a Raman shift of x = 700 and 1400 is equal. The normalisation factor is based just on this section as much greater variations arise in spectra outside of this range; however the same multiplicative factor was used on the entire spectrum for each sample. The normalisation process appeared to align the spectra intensities well. The actual area under the whole curve varied between 1.6 and 1.9.

• The data was smoothed with a Gaussian window function (a weighted linear filter) to smooth out some of the noise effects. The weights used were [0.0146 0.0831 0.2356 0.3333 0.2356 0.0831 0.0146 ]. Due to the measurement techniques used, we expected the spectra to be locally smooth, and this procedure helped to reduce the measurement noise seen in the data.

• A mean spectra for each patient is then produced which can be used for the analysis.

### Classification

Raman spectra are high-dimensional data sets. Each spectra contains 1200 Raman shift intensities. We wanted to be able to distinguish between cancer and non-cancer (respiratory) samples, from the mean spectra for each patient. Often it is useful to reduce the dimensionality of the data, and here we considered 3 ways of doing this: (1) by choosing the 25 best features from the 1200 data points using a two sample t-test to identify good features for the binary classification task, (2) using principal component analysis (PCA), to map the data to a lower dimensional space, here with 25 components, and (3) using a combination of the two, first choosing the 100 best features, and then PCA to reduce to 25. For discrimination between cancer and non-cancer cohorts LDA was performed on the reduced spectra. To evaluate performance, ten-fold cross-validation was performed, 100 times and an average performance calculated to avoid bias in the partition.

### Genetic algorithm

The mean spectra were provided as input sequences to the Implicit Context Representation Cartesian Genetic Programming algorithm (IRCGP)[14,15]. IRCGP uses evolutionary computing methodology to learn classifiers that are capable of distinguishing between data classes. Induced classifiers take the form of programmatic expressions applied to particular offsets within the input data sequences. These expressions are composed from a set of simple mathematical functions. Both the choice and connectivity of the functions, and the choice of offsets used within the input sequences, are determined by the algorithm's evolutionary process. The input sequences were divided equally into training and test sets. To prevent over-learning, training of the classifiers was stopped once classification accuracy of the test sequences started to fall.

## Results

All cancer patients agreed to be in the study. The mean age was 64.7 years, with 12 patients being male and 8 female. Twenty-one patients were recruited for the control group, 1 patient declined due to being on renal dialysis later that day and therefore 20 patients were used. The mean age for the control patients was 66.8 years, with 11 males and 9 females. Table 1 depicts the cancer types the patients of patients in this study. Table 2 depicts the illnesses the respiratory patients were suffering from.

**Table 1:** The cancer types present in this study.

**Type of Cancer**	**Number of patients**	**Lymph node involvement**
Squamous cell carcinoma	15	8

Malignant melanoma	1	1

Basal cell carcinoma	1	0

Unknown primary	1	1

Merkel cell tumour	1	1

Mucoepidermoid	1	0

**Table 2:** The respiratory disease in the control group of this study.

**Respiratory illness**	**Number of patients**
COPD and asthma	1

COPD alone	11

Asthma alone	4

Bronchiectasis with Kartagener's syndrome	1

Bronchiectasis	1

Bronchiectasis and asthma	1

Pulmonary fibrosis	1

**Figure 1:**
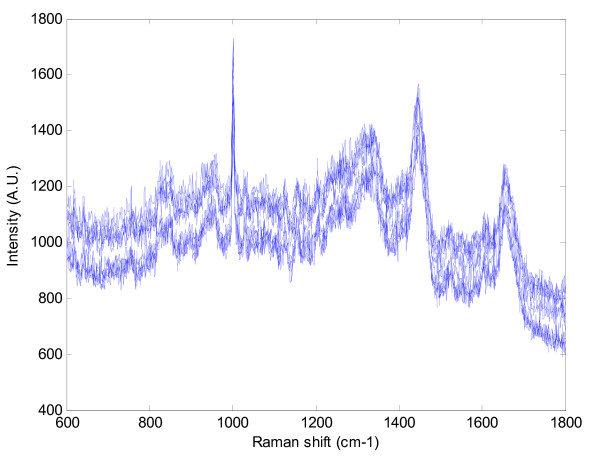
**An example set of ten Raman spectra obtained from the plasma sample of a patient with cancer**. The x-axis depicts the shift in wavelength (Raman shift) from the incident light.

**Figure 2:**
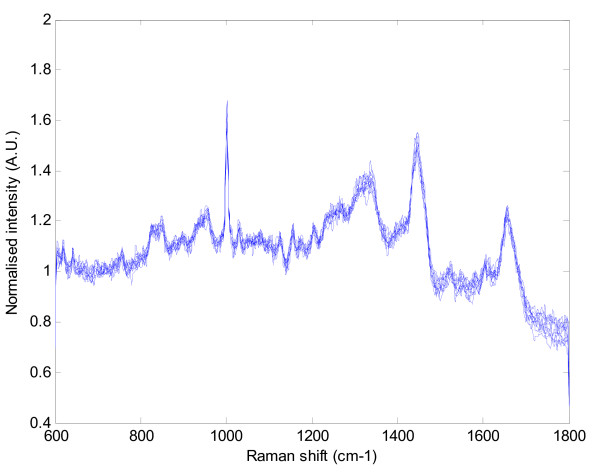
**An example set of ten Raman spectra from the plasma sample of a cancer patient after the data (figure 1) had undergone the normalisation process**.

The raw spectra show some variation in intensity levels (Figure 1), and the normalisation procedure outlined appeared to create well aligned spectra for each individual (Figure 2).

**Figure 3:**
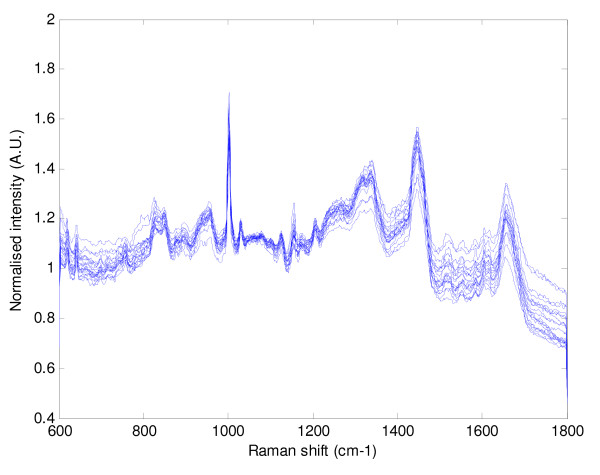
**The 20 mean spectra for the respiratory patients (non-cancer)**.

**Figure 4:**
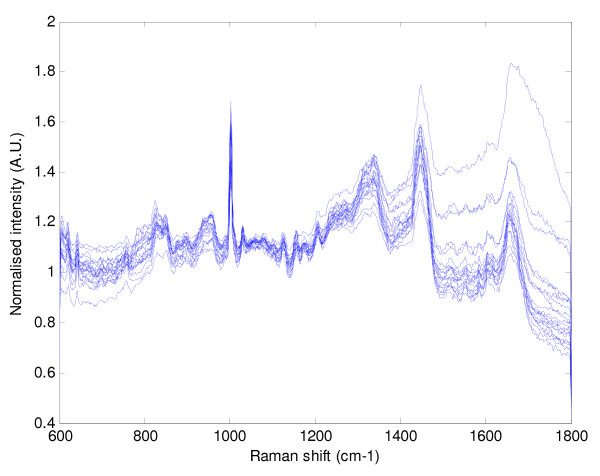
**The 20 mean spectra for the cancer patients**.

**Figure 5:**
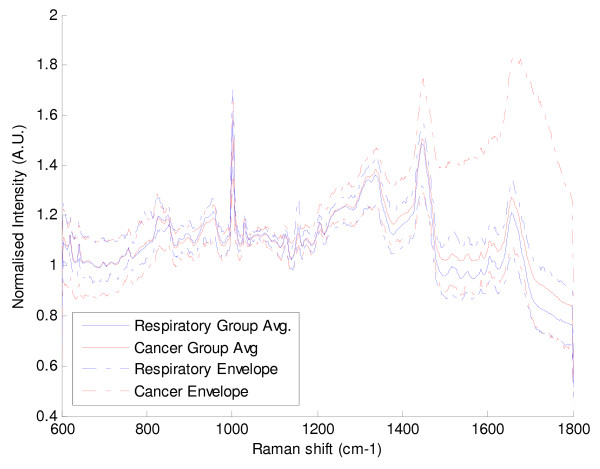
**Comparison of the means and range of cancer and respiratory (non-cancer) spectra**.

Figures 3 and 4 illustrate the graphical representation of mean Raman spectra for the 20 respiratory and the 20 cancer patients respectively. As can be seen the spectra are similar within each cohort and also between the cohorts. Figure 5 demonstrates just how similar the two sets of spectra are for the cancer and non-cancer (respiratory) groups.

Classification results for the four classification schemes used to classify the cancer and respiratory data are shown in Table 3. Conventional LDA approaches showed an accuracy of around 65% in the average of 100 runs of 10-fold cross validation. The best performance was 75% sensitivity and 75% specificity by the genetic evolutionary algorithm which obtained an accuracy of 83%. The result shown is from a single 10-fold cross validation, and the increase in performance is due to the way the learned rules combine various features in a non-linear way to predict the class labels. As an extra test, when the spectra were randomly assigned to Cancer/Non-Cancer labels, all the classifiers performed no better than random, adding support to the fact that we can distinguish between the cancer and non-cancer samples.

**Table 3:** The results obtained for the various classifier systems in discriminating between the cancer and non-cancer (respiratory) samples.

**Classifier Method**	**Accuracy (%)**	**Sensitivity (%)**	**Specificity (%)**
LDA on the best 25 features from the t-test	57.9	55.6	60.0

PCA-LDA (LDA on 25 principal components)	65.0	64.7	65.3

100 best > PCA 25 > LDA	65.8	64.9	66.7

Genetic Evolutionary Algorithm	83	75	75

**Figure 6:**
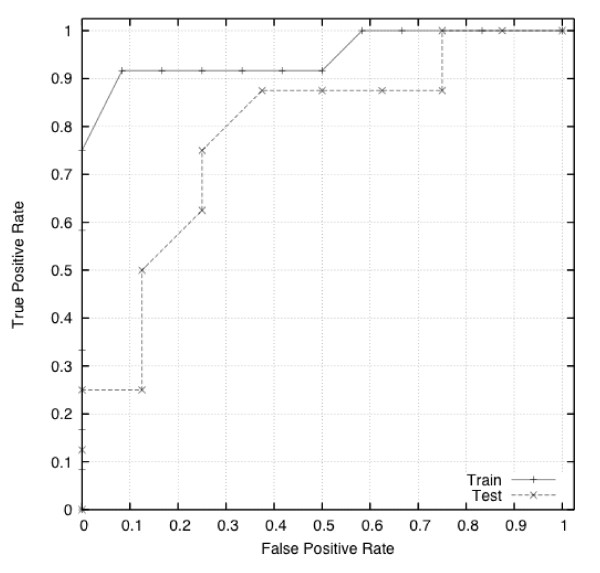
**The ROC curve illustrating the classification of cancer and non-cancer samples through the evolutionary algorithm**.

Figure 6 illustrates a receiver operating characteristic curve (ROC curve). This depicts the sensitivity versus 1 - the specificity. For the evolutionary algorithm used it can be seen that the most accurate algorithm produced 75% sensitivity with 75% specificity on the test data.

## Discussion

Respiratory patients were used as a control group for this study due to their similar ages, sex distribution and lifestyle risk factors, and also because of their co-morbid state. We therefore hoped to eliminate any differences between the cohorts as being due to the body's common response to a disease process.

We have demonstrated that using Raman spectroscopy and LDA analysis yields approximately 65% sensitivity and specificity for discrimination for cancer and non-cancer in peripheral blood samples. However, when the genetic programme was applied to the data, this increased sensitivity to 75% with 75% specificity.

In a similar study to ours Pichardo-Molina *et al *used Raman spectroscopy to compare serum samples from breast cancer patients and non-cancer patients [16]. They recruited 11 cancer patients (4 had metastatic disease) and 12 healthy controls to the study. Initially PCA was used to define wavelength differences between the 2 cohorts and ultimately to determine where differences were occurring in the native biochemistry. This latter proved to be unsuccessful due to the complex Raman 'fingerprint' and the multiple possible origins of peaks within the spectra. The authors achieved a 90% sensitivity utilizing LDA of the PCA scores and concluded that differences in Raman spectra were related to beta-carotene levels (peaks around 1155 and 1523); with generally lower values in the cancer cohort. Similar findings were reported by Li *et al*.2001 using a Raman system with incident wavelengths of 488 nm and 514.5 nm [17]. A large training set of 1022 samples containing various malignancies, healthy controls and other diseases was used, followed by 432 test samples. Results demonstrated between 70 and 100 per cent accuracies between cancer, non-cancer, and other illness groups, although their blood samples were stored at 4°C for up to 4 days making sample degradation a possible problem; especially if differing cohorts were run at different time points. In our study, LDA also highlighted differences in the Raman spectra at the 1150 region. The evolutionary algorithm discovered differences in between the cancer and non-cancer (respiratory) groups in the 1000 region of the spectra. This region is dominated by the phenylalanine band and may represent changes in the native state of this compound in the presence of cancer. It must be remembered that whilst compounds exhibit strong signals in certain regions of the spectra, they have many other peaks making determination of underlying biochemistry complex.

In a review of the accuracy of the conventional papanicolaou cervical smear test, Nanda and colleagues found sensitivity to vary between 30 to 87% and specificity from 86 to 100% [18]. Breast cancer screening is undertaken through 3 yearly mammography; Houssami and colleagues (2003) found approximate sensitivity for mammography to be 75% and specificity 88% [19]. Faecal occult blood testing has recently been introduced in the UK for individuals over 50 years of age. There is a large variation in sensitivity (12 to 82%) according to the region of the bowel affected; specificity is recorded at approximately 95% [20]. The sensitivity of our test in the current study is broadly similar to that of established screening programmes. The import factor is that specificity needs to be very high so no cancers are missed. The use of the ROC curve in this work allows a 'sliding scale' approach where increased sensitivity is matched by a decrease in specificity and vice versa, allowing a 'decision threshold' to be set.

There are limitations to our study especially the sample size which makes extrapolation onto populations difficult. This study was set out as a pilot project; head and neck patients were chosen yet the malignancies chosen were not only squamous cell carcinoma (90% of head and neck cancers). The next trial this group will undertake is to discriminate between malignancies (gastrointestinal, breast, etc) as well as a non-cancer cohort. Further work would then need to categorize patients regarding tumour load and spread. Patients used in these trails would need to be followed up after measurements have finished especially in the non-cancer group; as this may contain individuals with undiagnosed cancer or pre-malignant changes yet to be recorded. In this event what may seem erroneous results may well be accurate.

The ultimate aim is to be able to distinguish between cancers in the clinic setting; in this study the groups were quite coarse representations but the initial study was to solely to discriminate cancer from non-cancer using this methodology.

It may well prove to be the case that Raman spectroscopy could diagnose solid tumours from a peripheral blood sample in the future; however, a more feasible prospect is the ability to screen individuals at risk of cancer. Compared to cervical screening which is invasive, mammography which is undertaken in hospital, Raman blood analysis could be achieved at the local surgery. Along with screening, this test could be used as a diagnostic adjunct for those who attend the general practitioner with symptoms suggestive of malignant disease.

## Conclusion

This preliminary study has demonstrated the possible feasibility of using Raman spectroscopy in cancer screening and diagnostics of solid tumours through a peripheral blood sample. Further work needs to be carried out for this technique to be implemented in the clinical setting.

## Competing interests

The authors declare that they have no competing interests.

## Authors' contributions

ATH and AL undertook Raman spectroscopy on blood samples. CJN pre-processed the data and processed the linear discriminant analysis. MAL and SLS trained and tested the evolutionary algorithm. SEF, ASH and NC provided support with the samples. DAS provided support with the Raman system. JK, SEF, DAS, ASH and XY provided support with the sample collection and methodology. DPM-H provided support with the clinical application. All authors provided an editorial contribution.

All authors read and approved the manuscript.
